# Optimization of Saliva Collection and Immunochromatographic Detection of Salivary Pepsin for Point-of-Care Testing of Laryngopharyngeal Reflux

**DOI:** 10.3390/s20010325

**Published:** 2020-01-06

**Authors:** Young Ju Lee, Jiyoon Kwon, Sanggyeong Shin, Young-Gyu Eun, Jae Ho Shin, Gi-Ja Lee

**Affiliations:** 1 Department of Biomedical Engineering, College of Medicine, Kyung Hee University, Seoul 02447, Korea; younglee@khu.ac.kr; 2 Department of Chemistry, College of Natural Science, Kwangwoon University, Seoul 01897, Korea; jykwon@i-sens.com (J.K.); ssg0323@nanoentek.com (S.S.); 3 Department of Otolaryngology–Head and Neck Surgery, Kyung Hee University Medical Center, Seoul 02447, Korea; ygeun@khu.ac.kr

**Keywords:** salivary pepsin, collection, pre-processing, immunochromatographic strip, point-of-care testing

## Abstract

Salivary pepsin is a promising marker for the non-invasive diagnosis of laryngopharyngeal reflux (LPR). For reliable results regarding pepsin in saliva, it is critical to standardize the collection, storage, and pre-processing methods. In this study, we optimized the saliva collection protocols, including storage conditions, i.e., solution, temperature, and time, and the pre-processing filter for pepsin. Moreover, we prepared a simple immunochromatographic strip for the rapid detection of pepsin and evaluated its sensing performance. As a result, we selected a polypropylene (PP) filter as the pre-processing filter for salivary pepsin in low resource settings, such as those where point of care testing (POCT) is conducted. This filter showed a similar efficiency to the centrifuge (standard method). Finally, we detected the pepsin using gold nanoparticles conjugated with monoclonal pepsin antibody. Under optimized conditions, the lower limit of detection for pepsin test strips was determined as 0.01 μg/mL. Furthermore, we successfully detected the salivary pepsin in real saliva samples of LPR patients, which were pre-processed by the PP filter. Therefore, we expect that our saliva collection protocol and pepsin immunochromatographic strip can be utilized as useful tools for a non-invasive diagnosis/screening of LPR in POCT.

## 1. Introduction

Laryngopharyngeal reflux (LPR) is the backflow of gastric contents, such as food or stomach acid, into the larynx (voice box) and pharynx (throat), causing mucosal damage and several upper airway inflammatory disorders [1,2]. The symptoms of LPR usually include hoarseness, globus pharyngeus, chronic cough, dysphagia, throat clearing, and sore throat [3,4]. In general, the diagnosis of LPR has been based on laryngeal symptoms and laryngoscopic findings, including subglottic edema, erythema, posterior commissure hypertrophy, and thick mucus [5,6]. However, these methods lack sensitivity and selectivity for LPR detection [7]. Though ambulatory 24-h double-probe pH monitoring has been considered the gold standard for the diagnosis of LPR, it has some disadvantages, including invasiveness, high cost, and discomfort [8,9]. Therefore, it is necessary to develop an accurate, less costly, non-invasive diagnostic method for the diagnosis of LPR.

Saliva or sputum are biological fluids that are useful for new approaches to the clinical diagnosis and management of patients. It is known that they can reflect the physiological function and pathological conditions of the body [10,11]. In addition, saliva has many advantages, including easy and safe collection and inexpensive storage [11,12]. Therefore, saliva shows excellent potential for monitoring general health and disease [13]. Recently, it has become known that pepsin in saliva or sputum is a reliable diagnostic marker for LPR because it is produced only in the stomach and all refluxate contains it [6,8,14,15,16]. Several studies reported that pepsin could be a significant cause of laryngeal injury in nonacidic reflux [17,18]. In particular, mucous membranes of the laryngeal pharynx can be easily damaged by pepsin, compared to those of the esophagus [19]. Therefore, the detection of pepsin in the saliva can be utilized as a rapid, easy to perform, and cost-effective diagnostic method of LPR for point of care testing (POCT). Major challenges associated with saliva analysis include high viscosity and proteinaceous molecular assemblies that slow capillary flow through the device, variable flow rates, hindered transport of assay reagents, and aggregation of antigen detector molecules [20,21]. These matrix effects may interfere with diagnostic results and diminish the sensitivity of the immunoassay. However, appropriate sample pre-processing, including dilution, centrifugation, filtration, precipitation and extraction, can help to reduce or minimize the matrix effects [22]. Among them, freezing/centrifugation treatment was useful in minimizing the clogging effect of highly viscous mucins in saliva [19]. Yuksel et al. [23] reported the rapid salivary pepsin test for gastroesophageal reflux disease. However, they centrifuged saliva samples in a bench-top centrifuge, and then the supernatants were used for the pepsin test. Centrifugation cannot be used in limited-resource settings for point of care (POC) diagnostics. Besides, freeze-thawing may result in loss of quality of the protein analyte. There are different types of saliva sample collectors such as Salivette^®^ (Sarstedt AG & Co.), Quantisal^®^ (Abbott), and Certus^®^ (Abbott) [24]. However, they still have drawbacks in application to POCT because they require centrifugation. Saliva pre-processing procedures, including collection, storage, filtering, and transfer of POC diagnostics, are essential to achieving more sensitive, reliable, and reproducible results. However, there are no reports on the standardization of sample pre-processing procedures for salivary pepsin in POCT.

**Figure 1:**
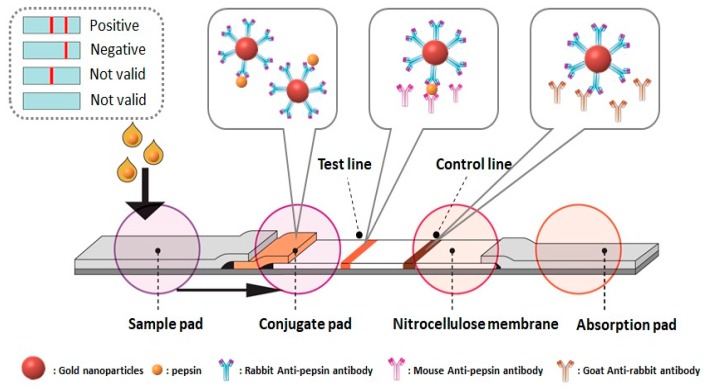
The schematic diagram for the detection process of the pepsin immunochromatographic strip. To start a test, we apply a sample containing the analyte (i.e., pepsin) to the sample pad, and it subsequently migrates to the other parts of the strip. At the conjugate pad, pepsin is captured by gold nanoparticle (AuNP)-antibody conjugate. This pepsin-binding conjugate reaches the nitrocellulose membrane and moves under capillary action. At the test line, the pepsin-binding conjugate is captured by another antibody (monoclonal) that is primary to pepsin. Excess AuNP-antibody conjugate will be captured at the control line by secondary antibody. The colorimetric intensity at the test line, which corresponds to the amount of pepsin in saliva samples, is captured using a digital camera and analyzed using ImageJ software. The appearance of color at the control line ensures that a strip is functioning correctly.

In this study, we optimized the storage conditions, including solution, temperature, and time, along with the pre-processing filters for POCT of salivary pepsin. Moreover, the performance of the selected filter was compared to that of the centrifuge, utilizing saliva samples of healthy volunteers (n = 5) and LPR patients (n = 8). Finally, we prepared a simple immunochromatographic strip for the rapid detection of pepsin and evaluated its sensing performance. The detection process of the pepsin immunochromatographic strip is shown in Figure 1. For real sample applications, the as-prepared immunochromatographic strip sensor was verified by determining the concentration of pepsin with saliva samples from healthy volunteers (n = 5) and LPR patients (n = 8). 

## 2. Materials and Methods

### 2.1. Materials and Chemicals

Citric acid (monobasic), 3,3′,5,5′-tetramethylbenzidine (TMB), tween-, bovine serum albumin (BSA), and goat anti-rabbit antibody were purchased from Sigma Chemical (St. Louis, MO, USA). We purchased the enzyme-linked immunosorbent assay (ELISA) kit for pepsin, anti-pepsin polyclonal antibody, and pepsin protein from Cloud-Clone Crop (Huston, TX, USA). The secondary antibody (horseradish peroxidase (HRP)-coupled secondary antibody) was purchased from Thermo Scientific (Rockford, IL, USA). The four kinds of commercially available syringe filters (0.45 μm pore size, 25 mm diameter), including nylon, polypropylene (PP), polyvinyl difluoride (PVDF), and polytetrafluorethylene (PTFE) were purchased from Whatman Inc. (Florham Park, NJ, USA). Artificial saliva was purchased from Kolmar Laboratories (Xerova—xerostomia saliva, Seoul, Korea). Gold nanoparticles (AuNPs; 30 nm diameter, stabilized suspension in 0.1 mM phosphate-buffered saline (PBS)) were purchased from Aldrich (Milwaukee, WI, USA). To fabricate the pepsin immunochromatographic strip sensors, we purchased mouse anti-pepsin antibody (monoclonal) and rabbit anti-pepsin antibody (polyclonal) from US Biological (Salem, MA, USA). Polystyrene microtiter plates were purchased from Corning (NY, USA). The sample and absorption pad (cellulose filter pad), nitrocellulose membrane (Hi-Flow 180), and conjugate pad (glass fiber pad) were purchased from Millipore (Bedford, MA, USA).

### 2.2. Optimization of Storage Conditions for Salivary Pepsin

To determine the optimal storage conditions such as solution, temperature, and time after collection of the saliva samples, we investigated the changes in the concentration of pepsin according to various storage temperatures, i.e., room temperature (RT), 4 °C and −20 °C, and storage times, 6 and 24 h. We also used various storage solutions, including citric acid (0.1 M, pH 2.5), acetic acid (0.01 M, pH 2.5), and PBS (0.1 M, pH 7.4). First, we added 100 ng/mL in PBS of pepsin (as mimicking salivary pepsin) into each storage solution and measured the pepsin concentration after 0 h (immediately) and 6 h at RT. After selecting the optimal storage solution as citric acid, we optimized the storage temperature and time as follows. The various concentrations (50, 100, and 200 ng/mL in PBS) of pepsin were added to 0.5 mL of 0.1 M citric acid, and these solutions were stored at different temperatures and times, including RT (6 and 24 h), 4 °C (6 and 24 h) and −20 °C (6 and 24 h). To quantify the concentration of pepsin, we coated each sample solution containing pepsin onto 96 well plates for 24 h. Each well was blocked with 5% BSA in PBS for 1 h at RT and washed with 1% BSA/PBS containing 0.05% Tween 20. The wells were incubated with a primary antibody (anti-pepsin antibody, 1:80) for 2 h at 37 °C. After washing, the wells were incubated with an HRP-coupled secondary antibody for 1 h at RT. A substrate and stop solution were introduced sequentially. The optical density (OD at 450 nm) of each well was determined within 30 min using a Synergy HT Multi-mode Microplate Reader (BioTek, Winooski, VT, USA).

### 2.3. Selection of the Pre-Processing Filter Using Volume and Protein Concentration Recovery Tests

To effectively remove the impurities in the field, we optimized the pre-processing filter for salivary pepsin. Four commercially available syringe filter membranes (nylon, PP, PVDF, and PTFE) were used for volume and protein recovery tests.

#### 2.3.1. Volume Recovery Test

To investigate the changes in the volume of solutions after passing through four syringe filter membranes, we utilized artificial saliva Xerova solution with and without BSA, as a model protein. Artificial saliva is mainly composed of NaCl, MgCl_2_, KCl, CaCl_2_·H_2_O, K_2_HPO_4_, sodium carboxymethyl cellulose, and D-sorbitol. First, two different volumes of each artificial saliva, 0.5 and 1.0 mL were mixed with 0.5 mL of 0.1 M citric acid. Pre-weighed artificial saliva mixtures with two different volumes, 1.0 and 1.5 mL, were passed through four different syringe filters, using a 10 mL syringe. Then, the filtered solution in the tube was weighed. Second, two different concentrations of BSA, 0.1 and 1 μg/mL, in artificial saliva, were mixed with 0.5 mL of 0.1 M citric acid. Pre-weighed artificial saliva mixtures with BSA went through the same procedure, with the filtered solution in the tube also being weighed. To verify the reproducibility, we performed all experiments in triplicate with a change in the experimenter. The data are expressed as the mean ± the standard deviation (S.D.) of the mean.

#### 2.3.2. Protein Concentration Recovery Test

To examine the change in protein concentrations after filtering, we mixed various concentrations of pepsin protein (0, 25, 50 and 100 ng/mL in PBS) with 0.5 mL of 0.1 M citric acid. Then, the mixtures were passed through the PP and PVDF filter membranes. The pepsin concentrations in each filtrate were analyzed using an ELISA assay. The recovered pepsin concentration was expressed as the percentages of the initial pepsin concentration. All experiments were performed in triplicate. 

#### 2.3.3. Performance Evaluation of the Pre-Processing Filter Using Saliva Samples

To evaluate the performance of the selected filter, we compared the pepsin levels in the saliva of LPR patients (n = 8), which were pre-processed by the PP filter and a centrifuge (as a reference method), utilizing a pepsin ELISA assay. The PP filter was selected as a pre-processing filter because it showed the highest volume and pepsin concentration recovery. The Ethics Committee of Kyung Hee University Medical Center (KMC IRB1432-01) approved this study and all participants signed informed consent. LPR patients (n = 8) and healthy volunteers (n = 5) were chosen based on clinical diagnostic criteria in the Department of Otolaryngology, Head and Neck Surgery. Subjects were instructed to collect saliva in the early morning before eating, drinking, or brushing their teeth [6]. To gather all the samples to test in a batch, we used 30 mL collection tubes containing 0.5 mL of 0.1 M citric acid [6,24,25]. Saliva samples were refrigerated at -80 °C and analyzed within two months of collection. To compare the performance of pre-processing in our new device with that of a centrifuge, we divided each sample in half. One was centrifuged at 14,000 g for 20 min at 4 °C, and the supernatant was harvested [6]. The other was placed into a disposable syringe and passed through a syringe filter, by a plunger, to obtain pre-processed samples. Pepsin concentrations in saliva samples that were pre-processed by centrifuge and the selected filter, respectively, were measured using a pepsin ELISA assay according to the manufacturer’s instructions. 

### 2.4. Preparation of the Immunochromatographic Strip for the Detection of Salivary Pepsin

#### 2.4.1. Preparation of the AuNP- Antibody Conjugates

To detect salivary pepsin, we used AuNPs as a colorimetric label. The gold nanoparticle (AuNP)-antibody conjugate was prepared by adding 50 μl of polyclonal antibody (rabbit anti-pepsin antibody, 200 μg/mL in PBS) into 5 mL of AuNP (30 nm diameter) solution adjusted to pH 9 with 0.1 M K_2_CO_3_ under vigorous stirring for 1h at RT, and then blocked with 3 mL of BSA (5% in PBS). After 10 min, the AuNP-antibody conjugate was collected by centrifuging (6000 rpm for 15 min) and washing with borate buffer (2 mM, pH 7.2) for three times. Finally, the obtained conjugate was suspended in 1% BSA/borate buffer (2 mM, pH 7.2) at 4° C until used. The variation in AuNP size after bonding with the antibody was confirmed using a UV-Vis spectrophotometer (Scinco, S-3100; Seoul, Korea). In addition, the colorimetric performance of the AuNP-antibody conjugate was evaluated using a sandwich-type ELISA assay.

#### 2.4.2. Pre-Processing Immunochromatographic Strip

The immunochromatographic strip system was composed of a sample pad, absorption pad, nitrocellulose membrane, and conjugate pad. The sample and absorption pads (i.e., cellulose filter pad) were pre-treated with 1% BSA/borate buffer (50 mM, pH 7.4) containing 0.05% Tween-20. The conjugation pad (i.e., glass fiber pad) was pre-treated with 2% BSA/borate buffer (50 mM, pH 7.4) containing 10% sucrose and 0.05% Tween-20. The nitrocellulose membrane was pretreated with PBS (10 mM, pH 7.2). After drying at 37 °C for 1h, the four different functional membrane pads were kept in a desiccator at RT until used to avoid moisture contamination. During the drying process, the evaporation occurs preferentially from the edge of the pad (edge effect). To reduce this effect, we did not use the edge for the strip.

#### 2.4.3. Fabrication of the Pepsin Immunochromatographic Strip

The pre-treated four different functional pads were used to prepare an immunochromatographic strip, as shown in Figure 1. The test line (7.0 mm^2^ in area) was formed by dispensing 2.0 μL of monoclonal antibody (mouse anti-pepsin antibody, 500 μg/mL in PBS) in nitrocellulose membrane (0.4 cm × 2.5 cm). The control line (7.0 mm^2^ in area) was formed by dispensing 2.0 μL of secondary antibody (goat anti-rabbit IgG antibody; 80 μg/mL in PBS) and separated from the test line by 1 cm. Both test and control lines were incubated for 1 h at 37 °C, blocked with 200 μl of BSA (5% in PBS) for 20 min at RT, washed with PBS, and then finally dried for 6 h at 37 °C. The conjugation pad (0.5 cm × 0.5 cm) was prepared by loading 20 μl of AuNP-antibody conjugate onto the entire pad and drying it for 2 h at 37 °C. The sample and absorption pads were prepared to 0.7 cm × 1.7 cm. The end of each pad was mounted to overlap with each other so that the sample solution flowed smoothly through the entire path (see Figure S1).

### 2.5. Analytical Performance of the Pepsin Immunochromatographic Strip

To examine the analytical performance of the immunochromatographic strips, we prepared artificial samples containing different concentrations of pepsin (0.01, 0.1, 0.5, 1.0, 2.5, 5.0 µg/mL in PBS) by mixing with 3% BSA, 0.05% Tween-20, and 1% methanol. Both BSA and Tween-20 were employed to prevent the nonspecific binding of proteins. The addition of a small amount of methanol may reduce the viscosity and the surface tension of the sample solutions, eventually resulting in improved sensor reproducibility. To test, we loaded 150 μL of each sample onto the sample pad of the prepared strip sensor and flowed through the path for 20 min. The colorimetric signal generated by the immuno-reaction was captured using a digital camera (FINEPIX-S9900W, Fujifilm, Japan) and analyzed using ImageJ software (NIH, Bethesda, MD, USA).

### 2.6. Real Sample Tests

Both the LPR patients (n = 8) and healthy volunteers (n = 5) were chosen based on clinical diagnostic criteria in the Department of Otolaryngology, Head and Neck Surgery. Their median age was 47 years; range 26–64 years. The saliva samples were collected and pre-processed using the same procedure. As described in Section 2.3.3, they were placed into a disposable syringe and passed through the PP syringe filter, by a plunger, to obtain pre-processed samples. The pre-processed saliva samples were used for the performance test of the pepsin immunochromatographic strip sensor.

## 3. Results and Discussion

### 3.1. Optimization of Storage Conditions including Solution, Temperature, and Time for Salivary Pepsin

Human saliva is a clear, slightly acidic (pH 6.0 to 7.0), and complex biofluid that is composed of water (99%), proteins (0.3%), and inorganic substances (0.2%) [26]. Saliva has been demonstrated to be a promising body fluid for the diagnosis of various diseases, including cancers [27], viral diseases [28], and autoimmune diseases [29]. The main advantage of saliva as a diagnostic tool is that its collection is easy and noninvasive, thereby significantly alleviating the subject’s discomfort as compared to blood collection. Therefore, salivary diagnostics are drawing particular attention in the fields that utilize POCT, as well as clinical applications for monitoring diseases frequently and easily, along with predicting posttreatment outcomes [13]. However, salivary constituents vary depending on the harvesting method and the degree of salivary flow [12]. Therefore, we standardized the storage methods, including solution, temperature, and time, for salivary pepsin.

**Figure 2:**
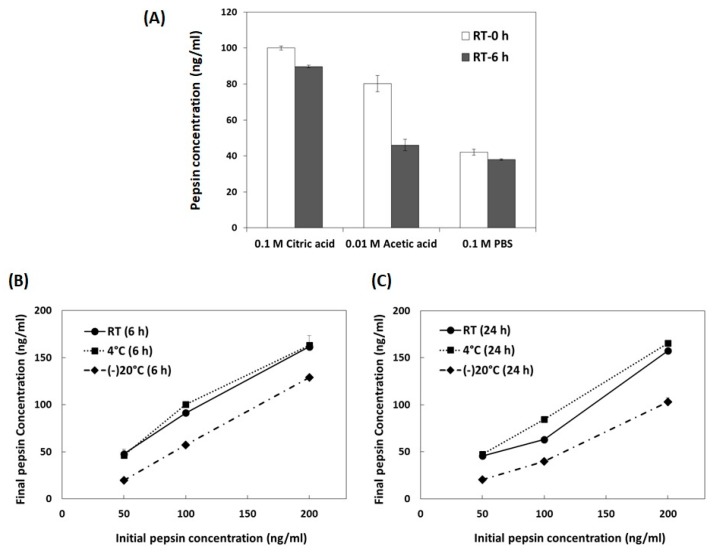
The effects of storage conditions including (**A**) solution, (**B**) temperature, and (**C**) time on the concentration of pepsin. The data are expressed as the mean ± the standard deviation of the mean.

First, we examined the stability of pepsin in various storage solutions, including citric acid (0.1 M, pH 2.5), acetic acid (0.01 M, pH 2.5), and PBS (0.1 M, pH 7.4), at RT. As shown in Figure 2A, the concentration of pepsin significantly decreased in acetic acid (80.2 ± 1.6 ng/mL for 0 h, 46.0 ± 0.4 ng/mL for 6 h) and PBS (42.1 ± 1.2 ng/mL for 0 h, 38.0 ± 0.7 ng/mL for 6 h). In particular, pepsin levels decreased by half as soon as it was added to the PBS. Citric acid was the most stable solution for pepsin, even after 6 h at RT (89.7 ± 3.1 ng/mL). Pepsin is the primary proteolytic enzyme of the digestive system and it shows maximal activity at a pH of 2.0 [30]. Kim et al., [31] reported that citric acid was a better alternative in the preparation of acidic pepsin solutions from the viewpoints of user safety and parasite survivability. It seemed that citric acid could keep pepsin stable and preserve its activity. As a result, citric acid was determined to be the best storage solution for pepsin. 

Second, we examined the effects of storage temperatures (RT, 4 °C, and −20 °C) and time (6 and 24 h) on pepsin levels. As shown in Figure 2B, pepsin after 6 h of storage at both RT and 4 °C showed good stability at 91.5 ± 0.7% and 100.2 ± 0.5%, respectively, compared to the initial concentration (100 ng/mL). However, the concentration of pepsin stored frozen at −20 °C for 6 h decreased in half (57.5 ± 1.6% versus the initial concentration of 100 ng/mL). Figure 2C represents the stability of pepsin according to storage temperatures after 24 h of storage. In this case, only pepsin at 4 °C maintained its stability (84.5 ± 2.0% versus the initial concentration of 100 ng/mL). The concentration of pepsin after 24 h of storage at RT and −20 °C decreased to 63.0 ± 1.0% and 40.0 ± 0.3% of the initial concentration (100 ng/mL), respectively. It is known that the freezing-thawing process can cause denaturation, aggregation, and functional loss of proteins [32]. Cao et al., [33] reported that the freezing damage of proteins in aqueous solutions could be reduced by changing the buffer type and composition, as well as optimizing the freezing-thawing protocol. From our result, it seemed that pepsin, in citric acid, suffered significant damage by freezing-thawing. Therefore, it is recommended to store pepsin in citric acid at RT/4 °C for 6 h or at 4 °C for 24 h.

### 3.2. Selection of the Filter for the Pre-Processing of Salivary Pepsin in a POCT

**Figure 3:**
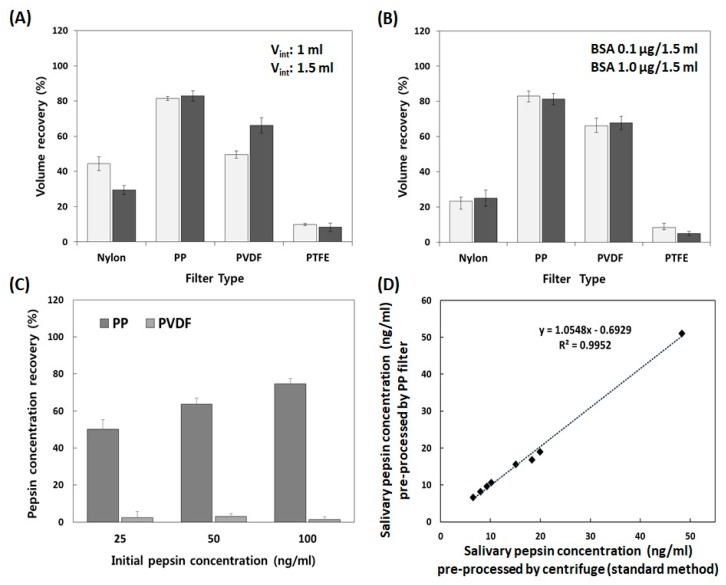
Comparison of the percent volume recovery from each syringe filter such as nylon, polypropylene (PP), polyvinyl difluoride (PVDF), and polytetrafluorethylene (PTFE), utilizing an artificial saliva solution (**A**) without and (**B**) with bovine serum albumin (BSA). (**C**) Shows the change in the concentration of pepsin after passing through a PP and PVDF filter, respectively. (**D**) Comparison of the concentration of pepsin in the saliva of laryngopharyngeal reflux (LPR) patients which is pre-processed with the PP filter and a centrifuge (as a standard method). The pepsin levels were measured utilizing a pepsin enzyme-linked immunosorbent assay (ELISA) assay.

Although a centrifuge is most commonly used in the laboratory to separate various impurities from biomarkers, it is likely not available in low resource settings where POCTs are conducted. Therefore, we attempted to select the optimal filter for removing impurities within saliva while minimizing the loss of pepsin. Considering the cost and the possibility of protein absorption, we chose four kinds of commercially-available syringe filters for pre-processing, including nylon, PP, PVDF, and PTFE. First, we performed the volume recovery test utilizing artificial saliva with and without BSA. As shown in Figure 3A, the PP filter showed the highest volume recovery (81.4 ± 1.2% for 1.0 mL and 82.9 ± 2.9% for 1.5 mL) in both starting volumes of 1.0 and 1.5 mL, respectively. The percent of volume recovery of a PVDF filter was 66.1 ± 4.3% for a starting volume of 1.5 mL and 49.630 ± 2.1% for 1.0 mL. Nylon possessed an intermediate volume recovery of 44.4 ± 4.0% for 1.0 mL, but the percent of volume recovery of nylon decreased to 29.4 ± 2.6% for 1.5 mL. In particular, the PTFE filter retained more fluid, resulting in the poorest volume recovery (9.8 ± 0.7% for 1 mL and 8.3 ± 2.4% for 1.5 mL). Figure 3B shows the results of volume recovery tests utilizing the same volume of artificial saliva with two different concentrations of BSA (0.1 and 1.0 µg/mL). As shown in Figure 3B, the PP filter showed the highest volume recovery at 82.9 ± 2.9% for 0.1 μg/mL and 81.2 ± 3.3% for 1 μg/mL. The PVDF filter had an intermediate volume recovery (66.1 ± 4.3% for 0.1 μg/mL and 67.8 ± 3.8% for 1 μg/mL). Nylon and PTFE showed the poorest volume recovery (nylon: 23.3 ± 2.2% for 0.1 μg/mL and 25.0 ± 4.5% for 1 μg/mL; PTFE: 8.3 ± 2.4% for 0.1 μg/mL, 4.9 ± 1.3% for 1 μg/mL). As a result, the PP filter showed the highest and the most reproducible volume recovery under all conditions.

Next, we examined the protein recovery of PP and PVDF filters utilizing various concentrations of pepsin. As shown in Figure 3C, the PP filter showed a good recovery capability (74.7 ± 1.5%) in the high concentration of pepsin (100 ng/mL). However, the PVDF filter did not recover well in any of the pepsin concentrations (2.9 ± 1.3%). Therefore, the PP filter was selected as a pre-processing filter for salivary pepsin, as it showed the best performance. These results demonstrated that the choice of filter for POCT might be critical to obtaining sensitive, reliable, and reproducible results.

To evaluate the performance of the selected filter, we compared the pepsin levels in the saliva of LPR patients, which were pre-processed by the PP filter and a centrifuge (as a reference method), utilizing a pepsin ELISA assay. Salivary pepsin in healthy volunteers (n = 5) was not detected in both samples which were pre-processed by the PP filter and a centrifuge. As shown in Figure 3D, the concentrations of salivary pepsin in all patients (n = 8) were similar in both the PP filter and centrifuge groups. It represented the good correlation (R^2^ = 0.9952) of salivary pepsin levels between the PP filter and a centrifuge as a standard method. These results demonstrated that the PP filter might be utilized as an effective pre-processing device for salivary pepsin in POCT.

### 3.3. Evaluation of the Performance of the AuNP-Antibody Conjugates

**Figure 4:**
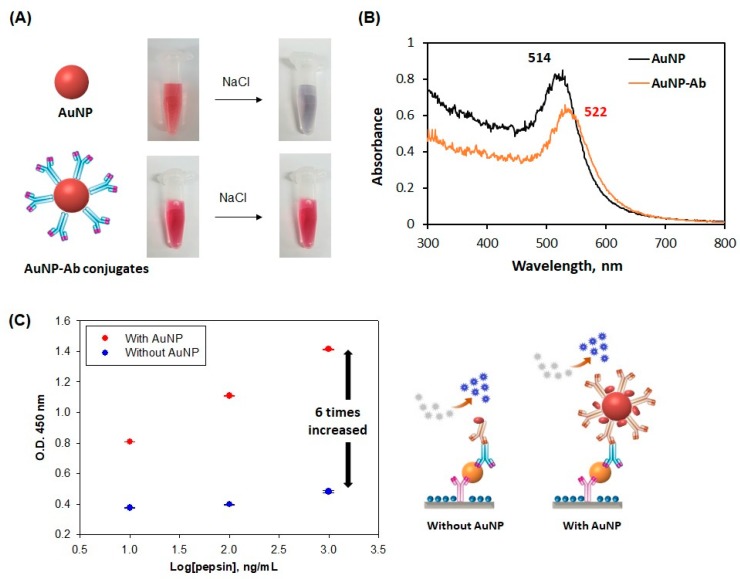
Evaluation of the optical performance of the gold nanoparticle (AuNP)-antibody conjugates. (**A**) Shows the stability of conjugates even after saltification; (**B**) shows the UV-Vis spectra of before and after the formation of conjugates, and (**C**) shows the result of the sandwich-type enzyme-linked immunosorbent assay (ELISA).

In general, AuNPs may be unstable alone in aqueous conditions, but they maintain a stabilized state by binding with proteins (e.g., antibody). Therefore, the AuNP-antibody conjugates kept a red color (i.e., non-aggregated) even after adding 10% NaCl. On the other hand, AuNP alone became midnight blue with the aggregation of AuNPs after adding NaCl (Figure 4A). To confirm the binding of the antibody to AuNPs, we measured the maximum absorption wavelengths of AuNPs (i.e., before and after conjugation) using a UV-Vis spectrophotometer. While the maximum absorption wavelength of the AuNP alone was observed at 514 nm, that of the AuNP-antibody conjugate was shifted slightly to 522 nm (Figure 4B). Figure 4C shows the ELISA results, exhibiting the effect of using AuNPs on the optical signal. As expected, the signal was amplified six times by employing AuNP-antibody conjugates, compared to the antibody alone. The use of AuNP-antibody conjugates may be effective in enhancing the sensitivity and detection limit of pepsin immunochromatographic strips.

### 3.4. Analytical Performance of the Immunochromatographic Strip for the Detection of Salivary Pepsin

**Figure 5:**
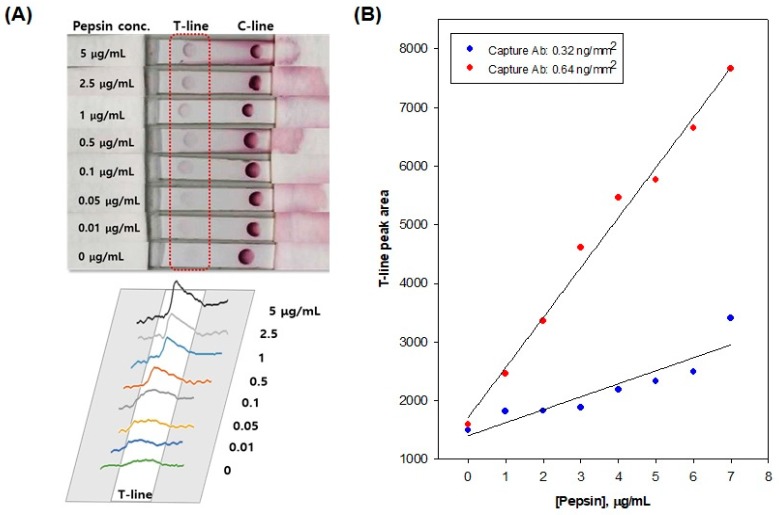
Evaluation of the analytical performance of the pepsin immunochromatographic strip sensor. Photographic images and signals analyzed by ImageJ software of immunochromatographic strips exposed to different levels of pepsin (**A**) and the calibration graph as a function of pepsin concentration (**B**).

To optimize the sensor performance of pepsin immunochromatographic strips, we prepared strips with two different amounts of monoclonal anti-pepsin antibody deposited on the test line, i.e., 0.32 and 0.64 ng/mm^2^. The analytical evaluation was performed using artificial samples containing different concentrations of pepsin, i.e., 0.0 (negative control), 0.01, 0.1, 0.5, 1.0, 2.5, 5.0 µg/mL in PBS. Figure 5A exhibits the photographic images of the immunochromatographic strips exposed to samples containing different levels of pepsin. The original image was converted to the peak intensity using ImageJ software. Figure 5B represents the calibration graphs, showing that the strips deposited with 0.64 ng/mm^2^ of the monoclonal anti-pepsin antibody have four times higher sensitivity than the strips with 0.32 ng/mm^2^. Furthermore, the resulting pepsin immunochromatographic strips enabled us to detect as low as 0.01 μg/mL of pepsin.

**Figure 6:**
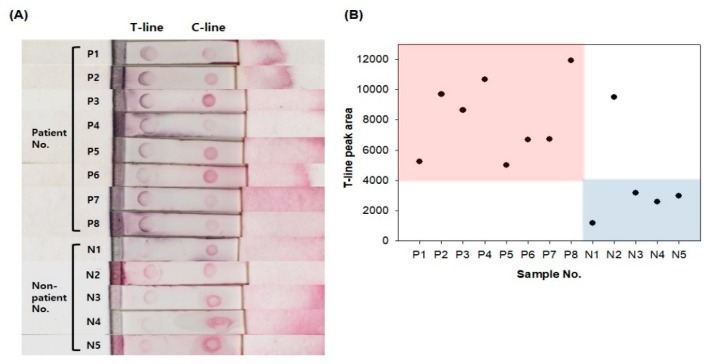
(**A**) Response of pepsin strip sensor in human saliva samples. (**B**) Colorimetric signal intensity graph in human saliva samples.

Pepsin in saliva has been receiving attention as a sensitive and specific marker for the diagnosis of LPR [2,18,34]. Na et al., [6] reported that the total levels of pepsin in saliva, collected upon waking, were significantly higher in the group of patients presenting with LPR symptoms. Therefore, the detection of pepsin levels in saliva can be utilized as a useful and convenient diagnostic tool for LPR. The analytical utility of the pepsin immunochromatographic strip sensor was verified by determining pepsin concentrations with the patient and healthy volunteer saliva samples. An apparent color change could be seen in the test line of the patient group compared to the non-patient group (Figure 6A). Additionally, as shown in Figure 6B, the graph can distinguish between non-patients and patients based on approximately 4000 peak areas. There is controversy among several studies regarding the presence of pepsin in healthy controls [6,34,35]. The studies that found pepsin in controls used clinically healthy controls, not confirmed with the gold-standard test [35]. It could mean that asymptomatic LPR patients might be included in controls. Therefore, we inferred that the high level of pepsin in N2 might be attributed to LPR episodes in asymptomatic LPR patients. The sensitivity and selectivity of our pepsin immunochromatographic strip should be further studied.

## 4. Conclusions

In summary, salivary pepsin is a promising marker for the non-invasive diagnosis of LPR. For best results in saliva-based LPR diagnostics, it is mandatory to standardize saliva collection, storage, and pre-processing methods for salivary pepsin. In this study, we optimized the storage conditions and pre-processing filter for salivary pepsin. To the best of our knowledge, this is the first report regarding the influence of storage conditions, including solutions, temperature, and time, on the stability of pepsin, as well as an effective filter for measuring salivary pepsin in POCT. In addition, we prepared an immunochromatographic strip with a good sensitivity to pepsin. As a result, our immunochromatographic strip sensors detected the salivary pepsin successfully in real saliva samples of LPR patients, which were pre-processed by the PP filter. Besides, we can distinguish between healthy control and LPR patients based on the color changes in the pepsin immunochromatographic strip. Therefore, our pre-processing protocol and immunochromatographic strip hold great promise, especially for non-invasive diagnosis of LPR in POCT.

## Supplementary Materials

The following are available online at https://www.mdpi.com/1424-8220/20/1/325/s1, Figure S1: Schematic of the immunochromatographic pepsin strip.
