# Supplementary material for: Optimization of Saliva Collection and Immunochromatographic Detection of Salivary Pepsin for Point-of-Care Testing of Laryngopharyngeal Reflux
Source: Sensors (Basel). 2020 Jan 6;20(1):325. doi: 10.3390/s20010325 (PMC6982828; doi:10.3390/s20010325)
Supplement: Supplementary file 1 [file sensors-20-00325-s001.pdf]

sensors

MDPI

Supporting Information

Article

Optimization of Saliva Collection and Immunochromatographic Detection of Salivary Pepsin for Point-of-Care Testing of Laryngopharyngeal Reflux

Young Ju Lee [1], Jiyoon Kwon [2], Sanggyeong Shin [2], Young-Gyu Eun [3], Jae Ho Shin [2,*] and Gi-Ja Lee [1,*]

[1] Dept. of Biomedical Engineering, College of Medicine, Kyung Hee University, Seoul 02447, Korea; younglee@khu.ac.kr

[2] Dept. of Chemistry, College of Natural Science, Kwangwoon University, Seoul 01897, Korea; jykwon@i-sens.com (J.K); ssg0323@nanoentek.com (S.S)

[3] Dept. of Otolaryngology–Head and Neck Surgery, Kyung Hee University Medical Center, Seoul 02447, Korea; ygeun@khu.ac.kr

* Correspondence: jhshin@kw.ac.kr (J.S); gjlee@khu.ac.kr (G.L)

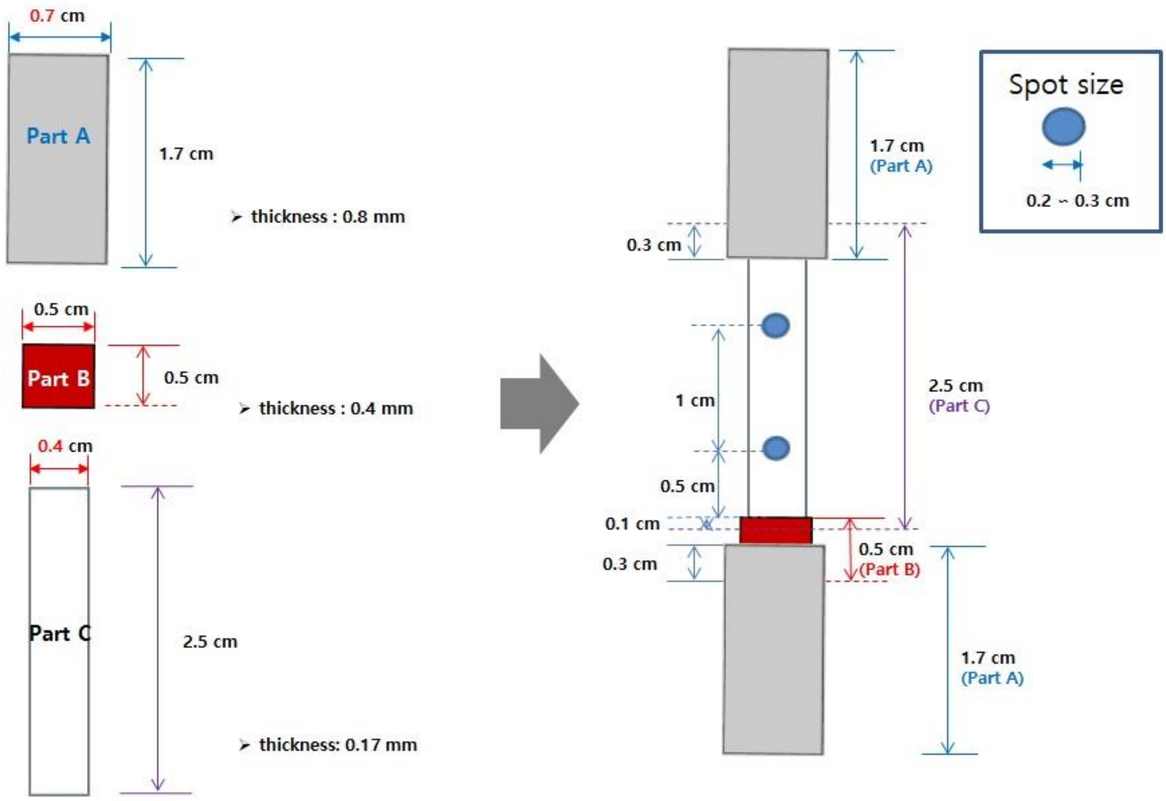

Figure 1. Schematic of immunochromatographic pepsin strip.
